# *In Vitro* and *In Vivo* Anti-Inflammatory Effects of TEES-10^®^, a Mixture of Ethanol Extracts of *Ligularia stenocephala* Matsum. & Koidz. and *Secale cereale* L. Sprout, on Gingivitis and Periodontitis

**DOI:** 10.3390/dj10080143

**Published:** 2022-08-02

**Authors:** Seungah Lee, In Hye Kim, Junkee Hong, Byung-Ju Jeon, Sung-Su Kim, Ji-Won Lee, Jin-Young Park, Seong-Ho Choi, Tae-Kyeong Lee, Jae-Kook Cha, Moo-Ho Won

**Affiliations:** 1 Naturesense Co., Ltd., Uiwang-si 16006, Korea; seungah.lee@famenity.com (S.L.); bj.jeon@famenity.com (B.-J.J.); 2 Famenity Co., Ltd., Uiwang-si 16006, Korea; inhye.kim@famenity.com (I.H.K.); jk.hong@famenity.com (J.H.); sungsu.kim@famenity.com (S.-S.K.); jiwon.lee@famenity.com (J.-W.L.); 3 Department of Periodontology, Research Institute of Periodontal Regeneration, Yonsei University College of Dentistry, Seoul 03722, Korea; jinyoungpark87@gmail.com (J.-Y.P.); shchoi726@yuhs.ac (S.-H.C.); 4 Innovation Research and Support Center for Dental Science, Yonsei University Dental Hospital, Seoul 03722, Korea; 5 Department of Food Science and Nutrition, Hallym University, Chuncheon 24252, Korea; tk_lee@hallym.ac.kr; 6 Department of Neurobiology, School of Medicine, Kangwon National University, Chuncheo 24341, Korea

**Keywords:** anti-inflammatory effect, anti-oxidative effect, *Ligularia stenocephala*, osteogenic differentiation, periodontal disease, *Secale cereale* L. sprout

## Abstract

Gingivitis and periodontitis are inflammatory disorders caused by dental plaque and calculus. These disorders often lead to tooth loss if not treated properly. Although antibiotics can be used, it is hard to treat them due to the difficulty in supplying effective doses of antibiotics to lesion areas and side effects associated with long-term use of antibiotics. In the present study, attempts were made to provide in vitro and in vivo evidence to support anti-inflammatory activities of TEES-10^®^, a mixture of ethanol extracts of *Ligularia stenocephala* (LSE) and *Secale cereale* L. sprout (SCSE) toward gingivitis and periodontitis by performing the following experiments. TEES-10^®^ with a ratio of 6:4 (LSE:SCSE) showed the best effects in both stimulating the viability and inhibiting the cytotoxicity. In in vitro experiments, TEES-10^®^ showed an ability to scavenge 2,2-diphenyl-1-picrylhydrazyl and superoxide radicals and remove ROS generated in periodontal ligament cells treated with lipopolysaccharide. TEES-10^®^ also enhanced the viability of stem cells from human exfoliated deciduous teeth and stimulated the osteogenic differentiation of deciduous teeth cells. In in vivo experiments using rats with induced periodontitis, TEES-10^®^ significantly decreased inflammatory cell infiltration and the numbers of osteoclasts, increased alveolar process volume and the numbers of osteoblasts, decreased serum levels of IL-1β and TNF-α (pro-inflammatory cytokines), and increased serum levels of IL-10 and IL-13 (anti-inflammatory cytokines). These results strongly support the theory that TEES-10^®^ has the potential to be developed as a health functional food that can treat and prevent gingival and periodontal diseases and improve dental health.

## 1. Introduction

Gingivitis and periodontitis are common dental inflammatory disorders caused by dental plaque and calculus that are formed due to poor dental hygiene. These lesions are susceptible to bacterial infection. If not treated properly, they can spread to the alveolar bone and eventually lead to tooth loss [1,2,3]. Periodontal disease is characterized by separation of teeth and gums due to an inflammatory response to microbial plaque. This feature occurs when a mixture of pathogenic and non-pathogenic bacterial species binds to the tooth surface and forms a biofilm [4]. Common periodontal pathogens include the anaerobic bacteria *Trepanema denticola* and *Porphyromonas gingivalis*, and the microaerobic bacteria *Aggregatibacter actinomycetemcomitans* [5,6]. Compounds are produced to trigger inflammatory responses [7]. An inflammatory response is induced by the direct action of LPS and an endotoxin which accelerates tissues damages by producing matrix metalloproteinases (MMPs), inflammatory cytokines [8,9] and reactive oxygen species (ROS) [10,11].

Of these mediators of tissue damage, ROS play major roles in tissue destruction. ROS are constantly produced in all living organisms [12,13]. They are produced in a huge amount from leukocytes during a bacterial infection in excess of removal capacity of the tissues [14,15]. Due to their high reactivity, ROS produced in gingivitis and periodontitis can destroy gingival and periodontal tissues [16,17]. By this mechanism of action, ROS also cause tissue damages in other inflammatory diseases such as atopic dermatitis and autoimmune arthritis [18].

As part of the treatment of gingivitis and periodontitis, mechanical removal methods such as scaling and root planning of the root surface are known as the gold standard [19]. However, scaling and root planning cannot completely remove the endotoxin that prevents the connective tissue from attaching to the root surface, and an amorphous smear is formed [20,21]. Therefore, systemic and topical antibiotics, topical drug delivery, host-controlled therapy, laser and other alternative therapies are used to support this, but all have limitations [22,23,24]. However, the use of antibiotics is limited due to the difficulty in supplying effective doses to lesion areas and side effects associated with their long-term use. Common side effects of using antibiotics include gastrointestinal disorders, tooth surface discoloration, loss of taste, disruption of oral bacterial balance and induction of resistant bacterial strains [25]. Thus, efforts have been made to search for therapeutic substances from biomaterials known to have fewer side effects [26,27,28]. Based on the roles of ROS in tissue damage and inflammatory conditions, much attention has been paid to substances with antioxidant activities [29,30].

*Ligularia stenocephala*, a perennial plant found in Korea, Japan, Taiwan, and China has been used as a vegetable. It is also used as a folk remedy for asthma and arthritis. Recent studies have shown that this plant has antioxidant, anti-inflammatory, anti-diabetic, and lipolytic effects [31,32,33]. *Secale cereale* L. is a kind of grain belonging to the gramineous plant and has spread to Europe and Northern Asia, in particular; it has been cultivated as a staple crop in Middle and Northern Asia [34]. It has been found that *Secale cereale* L. extracts have high antioxidant activities [35]. It has also been reported that a combination of probiotics and *Secale cereale* L. bran enhanced anti-inflammatory effects compared with probiotics or dietary fibers alone [36]; however, there are few studies on the effects of *Secale cereale* L. sprouts.

A previous clinical study [31] has shown that TEES-10^®^, a mixture of LSE and SCSE, can improve the symptoms of gingivitis and periodontitis [37]. In the present study, TEES-10^®^ was further investigated. The objectives of this study were: (1) to determine the ratio of LSE and SCSE that would lead to the highest efficacy, (2) to determine in vitro free radical scavenging and osteogenic activities of TEES-10^®^ with the optimal ratio of LSE to SCSE, and (3) to confirm therapeutic effect of TEES-10^®^ with the optimal ratio of LSE to SCSE in experimentally induced periodontitis in vivo using rats.

## 2. Materials and Methods

### 2.1. Preparing TEES-10^®^

TEES-10^®^ was uniquely produced and provided by Famenity Co., Ltd. (Uiwang, Gyeonggi, Korea) TEES-10^®^ is a mixture of ethanol extracts of LSE and SCSE, which was prepared according to a preparation process patented by Famenity Co., Ltd. [37]. It was provided by Famenity Co., Ltd. To determine the ratio of LSE:SCSE with the optimal effect, various preparations of TEES-10^®^ were made with the following ratios of LSE and SCSE, which were 1:9, 2:8, 3:7, 4:6, 5:5, 6:4, 7:3, 8:2 and 9:1.

### 2.2. Experiments Using Cells

#### 2.2.1. Determining the Optimal TEES-10^®^ Using Periodontal Ligament (PDL) Cells

To determine the TEES-10^®^ preparation with optimal efficacy, the above nine preparations of TEES-10^®^ were tested for the effects on the viability of PDL cells and the LPS-induced cytotoxicity of PDL cells.

PDL cells were supplied by Cell Engineering For Origin (CEFO™, Seoul, Korea) and cultured at 37 °C in a human periodontal ligament cell growth medium (CEFOgro™) containing 100 U/mL penicillin-100 μg/mL streptomycin (CEFOgro™) in a 5% CO_2_ incubator. The medium used in this experiment was replaced every two days, and the cells were cultured after reaching 90% confluency. We carried out PDL cell culture and cell viability measurement according to our previous method [37].

The cell viability of PDL cells was examined using the EZ-Cytox cell viability assay kit of Daeil Lab Service Co. (Seoul, Korea). PDL cells (8 × 10^3^ cells/well) used in this experiment were seeded in 96-well plates and incubated for one day. After removing the culture medium, the cells were incubated in the culture medium containing each of the TEES-10^®^ preparations (100 μg/mL) for 24 h. After adding the EZ-Cytox solution (10 µL) to each well, the cells were further incubated for 2 h and then the absorbance was measured at 450 nm with an Infinite M200 PRO NanoQuant microplate reader (TECAN, Männedorf, Switzerland).

To examine the cytoprotective effect of nine TEES-10^®^ preparations, the PDL cells were incubated with LPS (1 μg/mL) for 24 h. The cells were then incubated with each of the TEES-10^®^ preparations for 24 h and the cell viability was examined using the EZ-Cytox solution as above. In both experiments, the results were obtained from the five trials and presented as the mean ± SEM.

#### 2.2.2. Measuring 2,2-Diphenyl-1-Picrylhydrazyl (DPPH) Radical Scavenging Activity

TEES-10^®^ was tested for the activity to scavenge DPPH radicals (Sigma, St. Louis, MO, USA). DPPH (0.5 mM) were treated in the absence or presence of varying concentrations of TEES-10^®^ (12.5, 25, 50, 100, 200 or 400 μg/mL) for 30 min according to a previously described method [38]. The same experiment was done with 50 μg of L-ascorbic acid (Sigma), a positive control. The absorbance of the reactions was measured at 517 nm using the Infinite M200 PRO NanoQuant microplate reader (TECAN). DPPH was used after dissolving in DMSO. The activity of scavenging DPPH radicals was calculated using the following formula:

Inhibition (%) = {1 − [(A _Sample_ − A _Blank_)/A _Control_]} × 100

A _Sample_: absorbance in the presence of TEES-10^®^.

A _Blank_: absorbance in the absence of TEES-10^®^.

A _Control_: absorbance in the presence of L-ascorbic acid.

#### 2.2.3. Measuring Superoxide Radical Scavenging Activity (SOD-like Activity)

TEES-10^®^ was tested for the activity to scavenge superoxide radicals (O_2_∙^−^) according to the manufacturer’s protocol using the SOD assay kit of Dojindo Molecular Technologies, Inc. (Rockville, MD, USA). Briefly, TEES-10^®^ was dissolved in 200 µL of a working solution (which contains xanthine) provided by the manufacturer to varying concentrations (12.5, 25, 50, 100, 200 or 400 μg/mL), mixed with 20 μL of an enzyme working solution (which contains xanthine oxidase) and reacted at 37 °C for 20 min. The same experiment was done with 12.5 μg of L-ascorbic acid, a positive control. The absorbance of the reactions was measured at 450 nm. The SOD-like activity was calculated using the following formula:

SOD-like activity (%) = [(A _Blank 1_ − A _Blank 3_) − (A _Sample_ − A _Blank 2_)]/ (A _Blank 1_ − A _Blank 3_) × 100

A _Blank 1_: absorbance of the reaction containing xanthine (X) and xanthine oxidase (XO).

A _Sample_: absorbance of the reaction using TEES-10^®^ + X + XO.

A _Blank 2_: absorbance of the reaction containing X only.

A _Blank 3_: absorbance of the reaction containing X, XO and SOD.

#### 2.2.4. Measuring ROS Production from PDL Cells

The effect of TEES-10^®^ on the generation of reactive oxygen species (ROS) in the LPS-treated PDL cells was examined. We carried out 2′,7′-dichlorofluorescein (DCF-DA) staining in accordance with a precedent study [39]. PDL cells (8 × 10^3^ cells/well) were cultured for 24 h and cultured further for 24 h in the media containing LPS (1 μg/mL) with or without TEES-10^®^ (50 or 100 μg/mL). The cells were washed with PBS and cultured for 30 min in the presence of 20 μM DCF-DA (Sigma). Thereafter, the cells were washed and suspended in I mL of PBS. The fluorescence of the reactions was measured using a fluorescence microplate reader (Glomax, Promega, Madison, WI, USA) with 485/20 excitation filter and 528/20 emission filter.

#### 2.2.5. Measuring the Viability of Stem Cells from Human Exfoliated Deciduous Teeth (SHED)

SHED cells were supplied by Cell Engineering For Origin (CEFO™) and cultured in a SHED growth medium (CEFOgro™) containing 100U/mL penicillin-100 μg/mL streptomycin (CEFOgro™) at 37 ℃. The cells were subcultured 4–6 times after reaching 90% confluency changing the media every 2 days. 

To examine the effect of TEES-10^®^ on the viability of SHED, SHED (5 × 10^3^ cells/well) were cultured for 24 h and cultured further in the medium containing varying concentrations of TEES-10^®^ (10, 20, 50, 100, 200 or 400 μg/mL). The cell viability was measured using the EZ-Cytox Cell viability assay kit (Daeil Lab Service Co., Seoul, Korea) as described above. 

#### 2.2.6. Measuring Osteogenic Differentiation of SHED by Alizarin Red S Staining

To induce osteogenesis in the SHED, SHED (3 × 10^3^/well) were cultured in the Human MSC Differentiation Medium Osteogenesis (CEFOgro™) in the absence or presence of TEES-10^®^ (6:4) (100 μg/mL) for 24 h. After removing the medium, the cells were stained with 1% alizarin red S (pH 4.2; Sigma) at room temperature for 30 min to detect the calcification according to a previously published method [40], washed with distilled water and examined for calcification under a microscope. In addition, the color intensity was quantitated. 

### 2.3. Experiments Using Animals

#### 2.3.1. Inducing Experimental Periodontitis (EPD) in Rats

Sprague-Dawley rats (male, 6 weeks old) were acclimated in stainless steel cages (2 rats per cage) for 1 week. The twenty-three rats were anesthetized by intraperitoneally injecting a mixture (1 mL/kg) of Zoletil (Virbac, Milan, Italy) and Rompun (Bayer, Milan, Italy) (4:1 *v*/*v*). The five rats were subjected to a sham operation (control group) and eighteen rats received the EPD operation by ligating the right mandibular first molar with a 3-0 suture. The ligated 3-0 suture was left on the right mandibular first molar of rats. The nine rats (vehicle group) were given the vehicle and another nine rats (TEES-10^®^ group) were given TEES-10^®^ (50 mg/kg) for 4 weeks, respectively. TEES-10^®^ was orally administrated to the rats for 4 weeks every day from week 1 after ligating the right mandibular first molar.

#### 2.3.2. Histologic Examination of EPD Region

All the animals were anesthetized by intraperitoneally injecting a mixture (1 mL/kg) of Zoletil (Virbac) and Rompun (Bayer) (4:1 *v*/*v*) and perfused transcardially with 0.1 M phosphate-buffered saline (PBS, pH 7.4) followed by 4% paraformaldehyde in 0.1 M phosphate-buffer (PB, pH 7.4). The mandible tissue inducing the periodontitis was excised and immersed in 10% paraformaldehyde in 0.1 M phosphate-buffer (PB, pH 7.4).

The mandible tissue samples from the rats were obtained by crossly trimming around the first molar teeth region, including gingival and mandibular tissues. The mandibular tissues were treated with a decalcifying solution including 24.4% formic acid, and 0.5 N sodium hydroxide for 48 h. The resulting decalcified tissues were fixed in 10% neutral buffered formalin for one day and then made into paraffin blocks using the automated tissue processor of Shandon Citadel 2000 (Thermo Scientific, Waltham, MA, USA) and the embedding center of Shandon Histostar (Thermo Scientific). From each paraffin block, five μm-thick sections were made using an automated microtome (RM2255, Leica Biosystems, Nussloch, Germany). These sections were stained with hematoxylin and eosin (H & E) according to previously established methods [41,42] with our modifications and examined using the light microscope of Model Eclipse 80i (Nikon, Tokyo, Japan).

#### 2.3.3. Histomorphometry

The degree of inflammation of the EPD region was graded from a score of 0 to 3, considering the inflammatory cell influx, alveolar process and cementum integrity as described in Table 1 [41,43]. In addition, the infiltrated inflammatory cell numbers on the gingival sulcus (cells/mm^2^ of gingival tissues), alveolar process volumes between the periodontal ligament and inner gingival limits, and the mean osteoclast and osteoblast cell numbers on the inner alveolar process surface (cells/mm of alveolar process surface) were measured and analyzed using a computer-assisted image analysis program (iSolution FL ver 9.1; IMT i-solution Inc., Vancouver, British Columbia, Canada), based on previously established methods [44,45]. 

#### 2.3.4. Measuring Blood Levels of Some Biomarkers

After the 4-week experiments, the rats were anesthetized and 10 mL of blood was drawn from the inferior vena cava. The anesthesia and sacrifice were conducted as described in Section 2.3.2. The sera were obtained from the blood samples and assayed for markers of liver and kidney functions [aspartate aminotransferase (AST), alanine aminotransferase (ALT), and blood urea nitrogen (BUN)] and inflammatory cytokines (IL-1β, TNF-α, IL-10, and IL-13) using the MILLIPLEX MAP Rat Cytokine/Chemokine Magnetic Bead Panel of Immunology Multiplex Assay (Millipore, Billerica, MA, USA).

### 2.4. Statistical Analysis

All results obtained in this experiment were presented as mean ± standard error of mean (S.E.M). Statistical significance between groups was tested using ANOVA followed by Tukey’s test as a post-hoc test with the level of significance set to *p* < 0.05. The statistical significance of the results of the histomorphometric analysis performed on EPD-induced rats was tested using ANOVA followed by the least-significant differences multi-comparison (LSD) test with the level of significance set to *p* < 0.05. The Kruskal–Wallis H test was performed if a significant variance was found in the Levene test. If a significant difference was found in the Kruskal–Wallis H test, the Mann–Whitney U (MW) test was performed with the level of significance set to *p* < 0.05.

## 3. Results

### 3.1. Selecting the TEES-10^®^ with Best Efficacy

**Figure 1:**
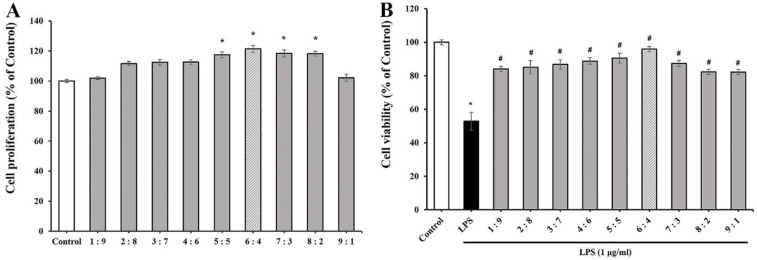
Effect of each preparation (100 μg/mL) on the viability of PDL cells (**A**) and LPS-induced cytotoxicity of PDL cells (**B**). The highest stimulation and inhibition is observed by the preparation of 6:4 ratio. Each bar represents the mean ± SEM (*n* = 5, respectively). * *p* < 0.05 as compared with control group; ^#^ *p* < 0.05 as compared with 1 μg/mL LPS-treated group.

In this study, an attempt was made to determine the ratio of LSE and SCSE to give the optimal efficacy. The results were shown in Figure 1. None of the TEES-10^®^ preparations reduced the viability of PDL cells. They rather stimulated the viability. The highest stimulation of 121.4% was observed at the LSE-SCSE ratio of 6:4 (Figure 1A). The viability of PDL cells was reduced to 50% by treatment with 1 μg/mL LPS. This reduced viability was inhibited by all the nine preparations. Here again, the highest inhibition was observed by the preparation of the 6:4 ratio (Figure 1B). Through the two experiments, TEES-10^®^ of 6:4 ratio was found to have the best efficacy, In the following experiments, the 6:4 preparation was used. 

### 3.2. Antioxidant Activities of TEES-10^®^

The antioxidant activities of TEES-10^®^ were evaluated by the three methods.

**Figure 2:**
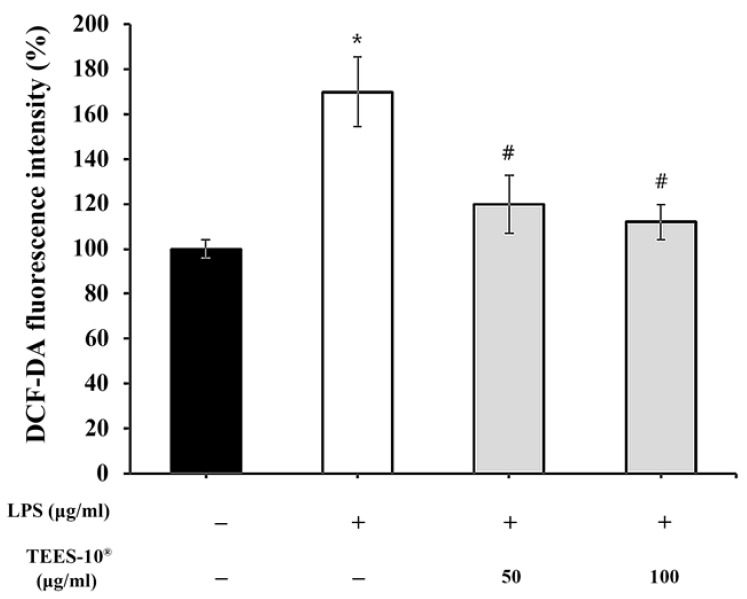
Effect of TEES-10^®^ on the production of ROS in the LPS-treated PDL cells. Increased ROS production in the LPS-treated PDL cells is inhibited by TEES-10^®^ dose-dependently. Each bar represents the mean ± SEM of fluorescence intensity (*n* = 5, respectively). * *p* < 0.05 as compared with control group; ^#^ *p* < 0.05 as compared with 1 μg/mL LPS-treated group.

The first was the inhibition of ROS generation in the LPS-treated PDL cells (Figure 2), which was measured by the fluorescence of DCF-DA. The ROS levels were increased up to 170% in the LPS-treated PDL cells compared to the untreated control. This increase of ROS production was inhibited by TEES-10^®^ dose-dependently. With 100 μg/mL, the production was inhibited to the control level. 

**Figure 3:**
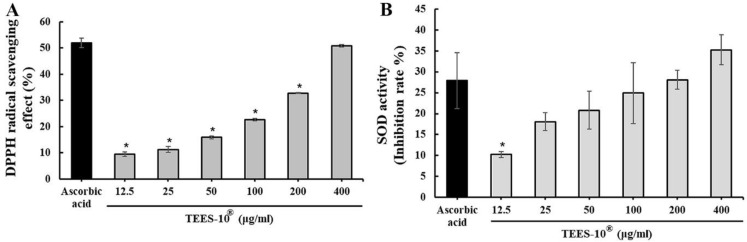
Activities of TEES-10^®^ scavenging DPPH (**A**) and superoxide (**B**) radicals. The activities of scavenging DPPH (**A**) and superoxide (**B**) radicals are dose-dependent. Each bar represents the mean ± SEM (*n* = 5, respectively). * *p* < 0.05 as compared with the activity of ascorbic acid.

The second was the scavenging of the DPPH radicals (Figure 3A). The activity of the scavenging DPPH radicals was dose-dependent from 12.5–400 μg/mL. The activity at 400 μg/mL was almost the same as that of 50 μg/mL of ascorbic acid. 

The third was scavenging superoxide radicals (SOD-like activity) (Figure 3B). The activity of scavenging superoxide radicals was also dose-dependent from 12.5–400 μg/mL. The activity at 400 μg/mL was even higher than that of 12.5 μg/mL of ascorbic acid.

### 3.3. Effects of TEES-10^®^ on Viability and Osteogenic Differentiation of Stem Cells from SHED

**Figure 4:**
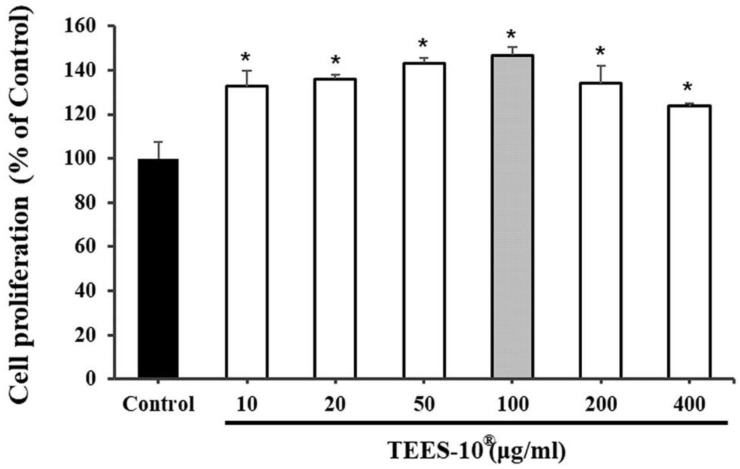
Effects of TEES-10^®^ on viability of stem cells from SHED cells. The highest stimulation is shown at 100 μg/mL. Each bar indicates the means ± S.E.M. (*n* = 5, respectively). * *p* < 0.05 as compared with control group with no TEES-10^®^.

TEES-10^®^ was examined for the osteogenic differentiation using SHED. First, TEES-10^®^ was tested on the viability of SHED (Figure 4). TEES-10^®^ did not affect the viability of these cells from 10 to 400 μg/mL and rather stimulated the viability in this range of the concentrations. The highest stimulation was observed at 100 μg/mL. Therefore, the effect on osteogenesis was tested with 100 μg/mL.

**Figure 5:**
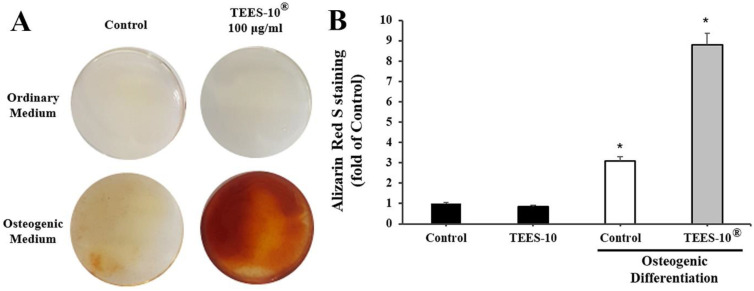
Effect of TEES-10^®^ on SHED cells. (**A**) Induction of osteogenesis by observing the increase of stained color intensity. The color intensity is significantly increased by TEES-10^®^ indicating that TEES-10^®^ can induce osteogenesis. (**B**) Quantitation of the staining intensity. The intensity is increased almost three-fold by TEES-10^®^. Each bar indicates the means ± S.E.M. (*n* = 3, respectively). Black bars: the results in the ordinary medium. * *p* < 0.05 compared to control group in ordinary medium.

As shown in Figure 5A, the induction of osteogenesis was confirmed by observing the increase in stained color intensity. The color intensity was significantly increased by 100 μg/mL of TEES-10^®^ indicating that TEES-10^®^ can induce the osteogenesis. SHED were not stained when the cells were cultured in the ordinary medium regardless of the presence of TEES-10^®^. In Figure 5B, the staining intensity was quantitated and the intensity was found to be increased almost three-fold by TEES-10^®^.

### 3.4. Anti-Inflammatory Effect of TEES-10^®^ on Experimental Periodontitis in Rats

**Figure 6:**
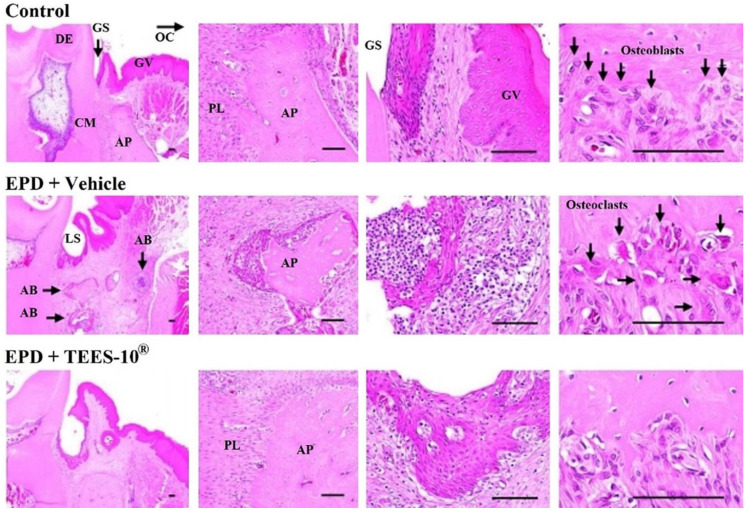
The effect of TEES-10^®^ in right mandible using H&E staining in rats. Noticeable infiltrations of inflammatory cells, increases in osteoclast cells and decreases in osteoblast cells are shown in EPD + vehicle group, but these inflammatory signs are significantly suppressed by TEES-10^®^. AB: abscess, AP: alveolar process, CM: cementum, DE: dentin, GS: gingival sulcus, GV: gingiva, LS = ligation site, OC: oral cavity, PL: periodontal ligament. Scale bars = 100 μm.

TEES-10^®^ was tested for the anti-inflammatory action using the experimental periodontitis in rats. The induction of periodontitis was confirmed in the H&E-stained tissue sections (Figure 6) which show marked and noticeable infiltrations of inflammatory cells, mainly neutrophils on the gingival tissues, increases in osteoclast cells and decreases in osteoblast cells together with alveolar process resorptions. These inflammatory signs were suppressed by TEES-10^®^ significantly. In Table 2, inflammatory cell numbers, alveolar process volumes and numbers of osteoblasts and osteoclasts were quantitated. TEES-10^®^ showed the significant suppressing effects on these inflammatory parameters. Table 2 also shows inflammatory scores that were estimated according to the criteria in Table 1. The score of periodontitis was 2.44 ± 0.73, which was decreased to 0.89 ± 0.33 by the treatment with TEES-10^®^.

**Figure 7:**
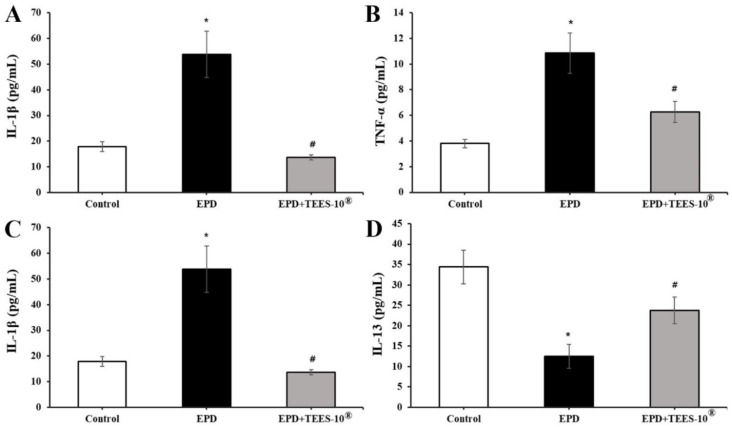
Effects of TEES-10^®^ on the serum levels of cytokines in rats with EPD. IL-1β (**A**), TNF-α (**B**), IL-10 (**C**), and IL-13 (**D**) are significantly increased in EPD + vehicle group, but they are significantly reduced by TEES-10^®^. Each bar represents the mean ± SEM (*n* = 5 in control and *n* = 9 in the other groups). * *p* < 0.05 as compared with control group; ^#^ *p* < 0.05 as compared with EPD group.

Anti-inflammatory action of TEES-10^®^ was also assessed by measuring the serum levels of cytokines associated with inflammation. In Figure 7A,B, IL-1β and TNF-α, which are pro-inflammatory cytokines, significantly increased in the EPD group but decreased significantly by treatment with TEES-10^®^. On the contrary in Figure 7C,D, the levels of IL-10 and IL-13, which are anti-inflammatory cytokines, significantly decreased in the EPD group but were increased significantly by TEES-10^®^. These results also support the anti-inflammatory action of TEES-10^®^.

The serum was also tested for AST, ALT, and BUN levels which are biomarkers related to liver and kidney functions. The levels of these markers were the same in the control, EPD and EPD + TEES-10^®^ groups (data not shown), suggesting that TEES-10^®^ does not affect liver and kidney functions. 

## 4. Discussion

In this study, in vitro and in vivo results indicate that TEES-10^®^, a mixture of LSE and SCSE, has a therapeutic potential for gingivitis and periodontitis. First, the ratio of LSE to SCSE that showed the highest efficacy was determined using nine preparations of TEES-10^®^ with ratios of LSE to SCSE ranging from 1:9 to 9:1. Of these nine preparations, the TEES-10^®^ with a ratio of LSE to SCSE at 6:4 showed the highest effects in stimulating the viability of PDL cells and inhibiting the LPS-induced cytotoxicity of these cells. Second, in in vitro experiments, the TEES-10^®^ could scavenge DPPH and superoxide radicals and remove ROS generated in PDL cells treated with LPS. The TEES-10^®^ could also enhance the viability of SHED stem cells of human teeth and stimulate the osteogenic differentiation of SHED cells. In in vivo experiments using rats with induced periodontitis, TEES-10^®^ suppressed inflammatory reactions significantly by decreasing inflammatory cell infiltration, increasing alveolar process volume, increasing osteoblasts and decreasing osteoclasts. TEES-10^®^ also decreased serum levels of IL-1β and TNF-α (pro-inflammatory cytokines) and increased serum levels of IL-10 and IL-13 (anti-inflammatory cytokines) in these rats.

The most common causes of gingivitis and periodontitis are dental plaque and calculus. Unless treated properly, these inflammatory disorders can damage the alveolar bone and ligaments supporting teeth [46]. If the lesion is infected with bacteria, the situation is exacerbated, eventually leading to loss of teeth [3]. In this pathologic condition, stem cells of teeth play important roles in repairing damaged tissues. These stem cells are rapidly differentiated into peripheral cells and osteogenic cells to actively regenerate lost tissues [47,48].

TEES-10^®^ (6:4) could effectively inhibit gingivitis and periodontitis. This action of TEES-10^®^ is assumed due to the following activities: (1) activities to stimulate the proliferation of cells of tooth supporting tissues and stem cells of teeth. This assumption was based on the findings that TEES-10^®^ stimulated the proliferation of PDL and SHED cells, which are periodontal ligament cells and stems cells of human deciduous teeth, respectively; (2) activities to stimulate differentiation of tooth stem cells. In addition to stimulating the proliferation, TEES-10^®^ also accelerated osteogenic differentiation. Several kinds of stem cells can be obtained from teeth and the surrounding tissues [49]. The SHED used in this study were obtained from the pulps of deciduous teeth or exfoliated natal teeth. The SHED have gained attention as a source of stem cells due to their high proliferation rates and osteogenic potential [50,51]. We observed a noticeable increase in the cell proliferation and the osteogenic differentiation level of SHED treated with TEES-10^®^. Since TEES-10^®^ promoted the proliferation and osteogenic differentiation of SHED, TEES-10^®^ may be expected to reinforce the function when we apply stem cells derived from deciduous teeth in tissue engineering after this; (3) ROS are well-known mediators causing tissue damage in inflammatory conditions. It has been reported that ROS are involved in tissue damage in periodontal inflammatory disorders [11,52,53]. The assumption that TEES-10^®^ is an efficient antioxidant is strongly supported by its ability to scavenge DPPH and superoxide radicals and remove ROS generated in LPS-treated PDL cells. The results of this study indicate that TEES-10^®^ has an excellent antioxidant effect and could effectively reduce the excessive oxidative stress caused by inflammatory reactions in PDL cells.

Consistent with the above in vitro results supporting its mechanism of actions [37], TEES-10^®^ (6:4) was observed to be able to efficiently suppress symptoms of experimentally induced periodontitis in rats. Macrophages stimulated by bacterial LPS product pro-inflammatory cytokines such as IL-1β and TNF-α [54]. Whereas a marked increase in the levels of IL-1β and TNF-α, which are mediators of strong inflammatory reactions involved in tissue destruction [55], was observed in the animal model of periodontitis compared to the control, a significant decrease in the levels of the same biomarkers was observed in the group orally administered TEES-10^®^. While the levels of IL-10 and IL-13, which are anti-inflammatory cytokines that regulate immune responses [56,57], decreased in the animal model of periodontitis, the levels of the same biomarkers increased in the group orally administered TEES-10^®^. These results indicate that orally administering TEES-10^®^ inhibits the activity of pro-inflammatory factors while restoring the activity of anti-inflammatory factors to reduce periodontal inflammation.

Both in vitro and in vivo results in this study further support the findings of a previous clinical study [37] showing that four-week administration of TEES-10^®^ to subjects with gingival problems can improve the gingival index (GI) and bleeding on probing (BOP) and remarkably reduce saliva levels of MMP-8 and MMP-9, which are enzymes that destroy connective tissues in inflammatory conditions. Taking the results of the present study and the previous study together, TEES-10^®^ is highly recommended as a functional food for preventing and treating dental inflammatory disorders. 

## 5. Conclusions

In the present study, TEES-10^®^ had a strong anti-oxidative effect by increasing the DPPH radical scavenging activity, increasing activity among scavenging superoxide radicals, and reducing ROS in LPS inflammation-induced PDL cells. In addition, TEES-10^®^ significantly promoted the osteodifferentiation of deciduous dendritic stem cells. Furthermore, in rats orally administered with TEES-10^®^ in an animal model of periodontitis, periodontitis symptoms and alveolar bone loss were inhibitory, inflammation-inducing cytokine levels in the blood decreased, and anti-inflammatory cytokine levels increased. Therefore, this study suggests the possibility that TEES-10^®^ can be developed as a safe natural material that can help prevent and improve periodontal health.

## Figures and Tables

**Table 1:** The Histological Scores of EPD used in this study.

Scores	Remarks
0	Absence or only a discrete cellular infiltration (inflammatory cell infiltration is sparse and restricted to the region of the marginal gingival), preserved alveolar process and cementum
1	Moderate cellular infiltration (inflammatory cellular infiltration present all over the insert gingival), some but minor alveolar process resorption and intact cementum
2	Accentuated cellular infiltration (inflammatory cellular infiltration present in both gingival and periodontal ligament), accentuated degradation of the alveolar process and partial destruction of cementum
3	Accentuated cellular infiltrate, complete resorption of the alveolar process and severe destruction of cementum
Max = 3	

EPD: Experimental periodontitis. EPD and related alveolar bone loss were induced by ligation placement around cervix of mandibular first molar teeth.

**Table 2:** The quantitative analysis of TEES-10^®^ effect on the experimental periodontitis (EPD) in rats.

Groups	Histological Scores (Max = 3)	Inflammatory Cell Numbers (cells/mm^2^ of Gingival Tissues)	Alveolar Process Volumes (%)	Osteoclast Cell Numbers (cells/mm^2^ of Alveolar Gingival Tissues)	Osteoblast Cell Numbers (cells/mm^2^ of Alveolar Gingival Tissues)
Control	0.20 ± 0.45	27.20 ± 8.20	70.93 ± 6.50	9.60 ± 4.56	91.60 ± 15.65
EPD + Vehicle	2.44 ± 0.73 ^a^	414.89 ± 280.59 ^c^	36.17 ± 12.20 ^c^	38.00 ± 9.27 ^a^	18.33 ± 7.14 ^a^
EPD + TEES-10^®^	0.89 ± 0.33 ^a^^, b^	133.78 ± 50.35 ^c^^, d^	61.77 ± 5.39 ^c^^, d^	2.89 ± 3.89 ^b^	56.67 ± 12.49 ^a^^, b^

The histologic preparations in Figure 6 were analyzed with respect to the numbers of inflammatory cells, volume of alveolar process and numbers of osteoclasts and osteoblasts. The severity of inflammation was scored according to the criteria in Table 1. Each bar represents the mean ± SEM (*n* = 5 in control and *n* = 9 in other groups)). ^a^ *p* < 0.05 as compared with Control group by LSD test. ^b^ *p* < 0.05 as compared with EPD + Vehicle group by LSD test. ^c^ *p* < 0.05 as compared with Control group by MW test. ^d^ *p* < 0.05 as compared with EPD + Vehicle group by MW test.

## Data Availability

The data presented in this study are available on request from the corresponding author.
